# Experimental Demonstration of a Tunable Energy-Selective Gamma-Ray Imaging System Based on Recoil Electrons

**DOI:** 10.3390/s24123736

**Published:** 2024-06-08

**Authors:** Changqing Zhang, Liang Sheng, Zhaohui Song, Tianxing Da, Haoqing Li, Baojun Duan, Yang Li, Dongwei Hei, Qunshu Wang

**Affiliations:** 1 Department of Engineering Physics, Tsinghua University, Beijing 100084, China; zcq19@mails.tsinghua.edu.cn; 2 National Key Laboratory of Intense Pulsed Radiation Simulation and Effect, Northwest Institute of Nuclear Technology, Xi’an 710024, China; songzhaohui@nint.ac.cn (Z.S.); li-hq17@tsinghua.org.cn (H.L.);; 3 School of Nuclear Science and Engineering, Shanghai Jiao Tong University, Shanghai 200240, China; datianxing@sjtu.edu.cn

**Keywords:** gamma imaging, energy-selective imaging, recoil electron detection, spatial resolution

## Abstract

The domain of gamma-ray imaging necessitates technological advancements to surmount the challenge of energy-selective imaging. Conventional systems are constrained in their dynamic focus on specific energy ranges, a capability imperative for differentiating gamma-ray emissions from diverse sources. This investigation introduces an innovative imaging system predicated on the detection of recoil electrons, addressing the demand for adjustable energy selectivity. Our methodology encompasses the design of a gamma-ray imaging system that leverages recoil electron detection to execute energy-selective imaging. The system’s efficacy was investigated experimentally, with emphasis on the adaptability of the energy selection window. The experimental outcomes underscore the system’s adeptness at modulating the energy selection window, adeptly discriminating gamma rays across a stipulated energy spectrum. The results corroborate the system’s adaptability, with an adjustable energy resolution that coincides with theoretical projections and satisfies the established criteria. This study affirms the viability and merits of utilizing recoil electrons for tunable energy-selective gamma-ray imaging. The system’s conceptualization and empirical validation represent a notable progress in gamma-ray imaging technology, with prospective applications extending from medical imaging to astrophysics. This research sets a solid foundation for subsequent inquiries and advancements in this domain.

## 1. Introduction

Gamma-ray imaging is an indispensable technology with significant applications across various domains. Its strong penetrating power makes it ideal for transmission imaging, which is utilized in fields such as medical diagnostics [1,2,3] and nuclear security [4,5,6,7]. Gamma rays emitted from operational radiation sources, including those in inertial confinement fusion (ICF) facilities, provide valuable insights into their functioning [8,9]. Selective visualization of gamma rays at specific energy levels is crucial for enhancing contrast and diagnostic effectiveness in these applications. However, traditional detectors lack the flexibility to adjust energy windows, which restricts detailed spectral analysis and the resolution of complex spectra.

The advancement of energy-selective imaging techniques is particularly critical for ICF experiments, where gamma rays carry unique energies and convey specific physical information [10]. For example, gamma-ray lines at 16.75 MeV indicate total fusion yield; 15.58 MeV, fuel areal density; and 4.44 MeV, carbon ablation areal density [11,12,13]. Precise differentiation of these energy levels is essential for refining ICF devices and evaluating the symmetry of fusion materials.

This article presents a pioneering approach to energy-selective gamma-ray imaging, leveraging the detection of recoil electrons for high spatial resolution and tunable energy discrimination. Inspired by Compton magnetic spectrometers, our system integrates a conversion target to transform gamma rays into electrons. The ensuing recoil electrons, generated through interactions of gamma rays with the converter, are captured by a purpose-built double-bend imaging system. Through energy and trajectory analysis, the energy of the incident gamma rays is deduced, enabling energy-selective imaging. The manuscript also provides an experimental evaluation of this system, detailing its design and presenting a comprehensive analysis of the experimental data. This demonstrates the system’s proficiency in imaging performance and energy selection.

The article is structured as follows: Section 2 details the conceptual design of the energy-selective gamma imaging system. Section 3 elucidates the experimental setup. Section 4 presents the experimental results and discussion. Conclusions from the study are articulated in Section 5.

## 2. Conceptual Design

**Figure 1:**
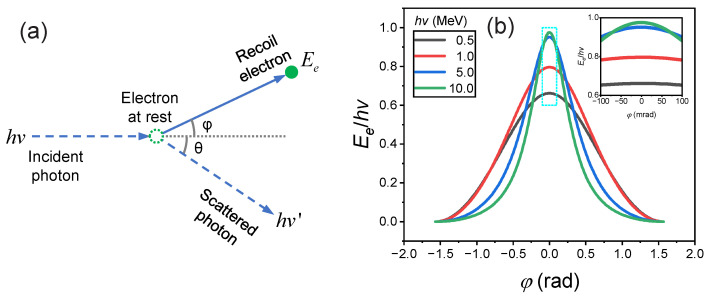
(**a**) Schematic display of Compton scattering. (**b**) Energy distribution of Compton recoil electron: dependency on photon energy and scattering angle φ.

The energy-selective imaging system proposed in this study operates on a principle similar to that of a Compton spectrometer [14,15,16], both of which are based on Compton scattering. This phenomenon is predominant when MeV gamma photons interact with materials of a low atomic number [17]. Figure 1a illustrates the process of Compton scattering, where incident gamma photons interact with electrons, resulting in the production of recoil electrons.

A direct correlation exists between the energy of the recoil electron $E_{e}$, its scattering angle φ, and the energy of the incident photon hν, as detailed in Equation (1). Figure 1b shows the relationship between the normalized electron energy $E_{e}/h\nu$ and the scattering angle φ for photon energies ranging from 0.5 to 10 MeV. For scattering angles |φ| below 100 mrad, the normalized electron energy remains relatively constant. This implies a strong correlation between the energy of the forward-scattered Compton recoil electron and that of the incident photon in scenarios of small-angle scattering. Using forward-emitted Compton recoil electrons, it is possible to determine not only the energy spectrum of the incident gamma rays but also to deduce the site of interaction between the gamma rays and the conversion target, thus facilitating indirect gamma-ray imaging.

(1) $$E_{e}=\frac{2E_{\gamma }\left(\frac{E_{\gamma }}{m_{e}c^{2}}\right)}{1+2\left(\frac{E_{\gamma }}{m_{e}c^{2}}\right)+{\left(1+\frac{E_{\gamma }}{m_{e}c^{2}}\right)}^{2}\tan^{2}\left(\varphi \right)}$$

**Figure 2:**
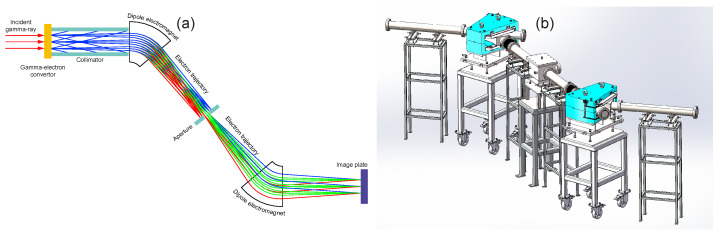
Design of an energy-selective gamma-ray imaging system based on recoil electron: (**a**) Schematic of gamma photon and Compton electron motion. The trajectories of electrons with varying energy levels, differentiated by color. Electrons with a greater energy spread, marked in red and blue, are obstructed by the aperture, thereby preventing their further transport and achieving energy selection. (**b**) Schematic of system mechanical structure.

A novel imaging system has been introduced for gamma-ray energy-selective imaging predicated on the detection of recoil electrons [18], as illustrated in Figure 2a. This system is constructed around three integral components:

1. Radiation Conversion Target, which utilizes a Beryllium foil to facilitate the interaction between gamma rays and electrons, thereby generating recoil electrons;
2. Achromatic Point-to-Point Imaging Structure, featuring an odd-symmetric configuration of two dipole electromagnetic coils designed to accurately image recoil electrons across a spectrum of divergence angles and energy distributions, while also correcting for chromatic aberrations to maintain imaging clarity and precision;
3. Image Plate, which is responsible for capturing and recording the spatial distribution of electron positions, subsequently transforming this information into image data for further analysis and processing.

The conceptual design of the system has been meticulously developed, establishing the foundational framework for our research. However, the system’s performance needs empirical validation. Therefore, upon the system’s construction as depicted in Figure 2b, an experimental study was conducted to thoroughly evaluate its functionality and efficacy. In the subsequent sections, we will describe the experimental setup, detail the methodologies applied, and present the findings obtained from this study. This will offer a comprehensive assessment of the system’s operational capabilities and its potential applications.

## 3. Experimental Setup

**Figure 3:**
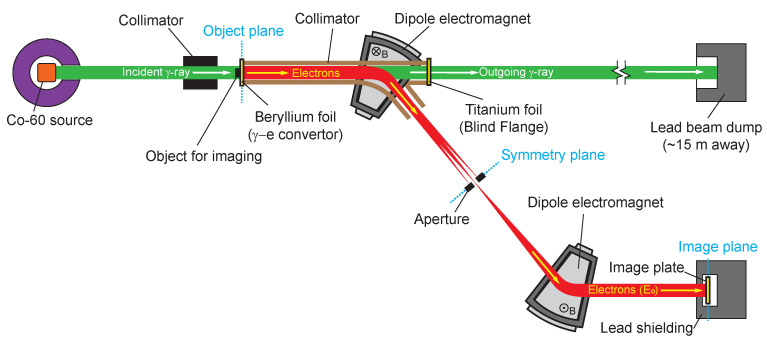
Schematic of the experimental setup.

The experimental setup is illustrated in Figure 3. A Co-60 gamma-ray source was used for calibrating the system. Each radioactive decay of the Co-60 source produces two gamma quanta with energies of either 1.173 MeV or 1.332 MeV, as reported in [19]. The gamma rays emitted from the Co-60 source traverse a tungsten collimator and strike the surface of a beryllium converter vertically, thereby generating electrons. As depicted in Figure 3, the converter is situated at the object plane. An object positioned in front of the converter reduces gamma-ray intensity, leading to changes in the electron intensity distribution. The image plate at the image plane captures these changes, thereby facilitating the indirect imaging of the object. The converter is situated approximately 1.5 m from the source’s center. The dose rate at the converter, measured to be approximately 49.84 R/min, has been calibrated using a PTW UNIDOS [20], with dosimetry accuracy within 2.5%. The system was exposed for a duration of 30 min to accumulate a substantial quantity of photons, ensuring the detection of a sufficient number of Compton scattered electrons for calibration purposes. The specific parameters of the gamma-ray beams incident on the converter are enumerated in Table 1.

The Compton recoil electrons, emitted anteriorly from the beryllium surface, are directed into a double-bend achromat imaging system. The tungsten collimator, located at the entrance of the first magnet, determines the scattering angle of the electrons. With a diameter of about 100 mm, it is precisely positioned to ensure a distance of approximately 1100 mm from its exit to the converter. This setup limits the angular dispersion of the recoil electrons entering the magnet to approximately 90 milliradians. (For an estimation of the energy distribution of Compton recoil electrons, see Figure 1b). The collimated electrons then enter a dipole magnet system, where those with a central energy $E_{0}$ are deflected by the first dipole magnet and subsequently focused at the symmetry plane. The central energy $E_{0}$ of an electron is correlated with the magnetic flux density *B* at the center of a dipole magnet. This correlation arises from the balance between the Lorentz force and the centripetal force in a magnetic field. The Lorentz force, which dictates the trajectory of charged particles within a magnetic field, is defined by the equation:

(2) $$\vec{F}=q\left(\vec{v}\times \vec{B}\right)$$

where $\vec{F}$ signifies the Lorentz force, *q* is the charge of the electron, $\vec{v}$ represents the electron’s velocity vector, and $\vec{B}$ denotes the magnetic field vector. This force also acts as the centripetal force responsible for the electron’s circular motion, as expressed by

(3) $$q\vec{v}\times \vec{B}=\frac{mv^{2}}{r}\hat{r},$$

where *m* is the electron’s rest mass, *v* is the velocity magnitude, *r* is the radius of the circular orbit, and $\hat{r}$ is the unit vector pointing toward the center of the orbit. For electrons with MeV-level energies that approach the speed of light, relativistic effects are essential to include in the calculations. The total energy *E* of a relativistic particle is encapsulated by

(4) $$E^{2}={\left(mc^{2}\right)}^{2}+{\left(pc\right)}^{2}.$$

In this formula, $E=\gamma mc^{2}$ is the total energy, $\gamma =1/\sqrt{1-\left(v^{2}\right)/\left(c^{2}\right)}$ is the Lorentz factor, and *c* is the speed of light. The relativistic momentum is given by p=γmv. The central energy $E_{0}$ of the electron, which is the kinetic energy in the relativistic framework, can be calculated as the difference between the total energy and the rest energy, $E_{0}=E-mc^{2}$. By integrating Equation (3) with Equation (4), the relationship linking the electron’s central energy $E_{0}$ to the central magnetic flux density *B* of the dipole magnet is derived as

(5) $$B=\frac{\sqrt{E_{0}\left(E_{0}+2m_{e}c^{2}\right)}}{cqR}.$$

Equation (5) reveals a one-to-one correspondence between the electron energy $E_{0}$ at the image plane and the central magnetic flux density *B* of the dipole magnet. When the central magnetic flux density of both magnets is unified at *B*, only recoil electrons with energy approximating $E_{0}$ can traverse to the system’s imaging plane, thus enabling aberration-corrected point-to-point imaging. Electrons exhibiting a greater energy dispersion, quantified by $\delta =\left(E_{e}-E_{0}\right)/E_{0}$, are deflected by an aperture located at the symmetry plane, thereby terminating their progression through the system and completing the energy selection process. By modulating the current through the magnets, the central magnetic flux density *B* of the dipole magnets can be adjusted, allowing for the imaging of recoil electrons with varying energies. This adjustment, in turn, indirectly supports energy-selective imaging of the incident gamma rays.

The spatial distribution of recoil electrons on the imaging plane is captured by a BAS-MS type image plate (IP), produced by Fuji Film Corporation. This image plate is subsequently scanned using a GE Amersham scanner, which offers a resolution of 100 μm, a dynamic range latitude of 5, and a sensitivity rating of 1000. Utilizing the calibration technique as detailed by Williams et al. [21], the signal intensity recorded on the imaging plate is translated into a measure of photon count per pixel, expressed in photo-stimulated luminescence (PSL) per pixel, through the application of Equation (3).

(6) $$\mathrm{PSL}={\left(\frac{G}{2^{16}-1}\right)}^{2}{\left(\frac{R_{\mu m}}{100}\right)}^{2}h\left(V\right)10^{\frac{L}{2}},$$

where G is the scanned image value, $R_{\mu m}$ is the spatial resolution, $h\left(V\right)$ is the sensitivity function and *L* is the dynamic range latitude.

**Figure 4:**
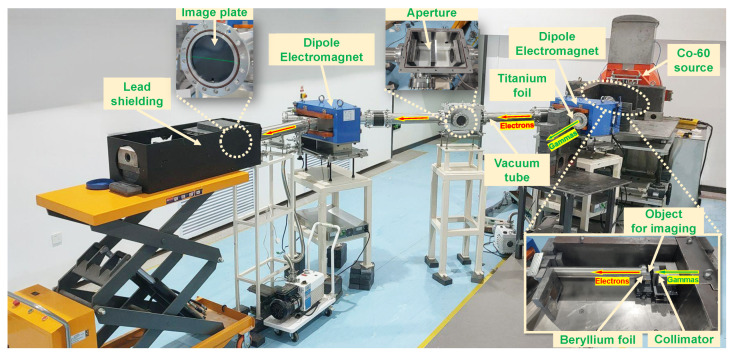
Assembly and experimental setup of the proposed imaging system.

Figure 4 displays the image of the fully assembled imaging system. The vacuum chamber’s pressure is consistently maintained below 0.1 Pa throughout the photographic processes. The 150-micrometer-thick beryllium foil fulfills two roles: it serves as a converter for transforming gamma rays into electrons and also functions as a sealant for the vacuum system. The system’s pivotal performance parameters include spatial resolution, energy resolution, and detection efficiency. Considering the gamma beam spot’s diameter, post-collimation, is about 40 mm with a nearly uniform distribution, we utilize the gamma-ray radiography technique to assess the system’s spatial resolution capabilities. As the Co-60 source emits gamma rays with two distinct energy levels, separate imaging for each energy can be performed by adjusting the magnet’s current to modify the central magnetic field. Combined with a quantitative analysis of the imaging plate’s output, this method enables an estimation of the system’s energy resolution and detection efficiency.

According to Lambert–Beer Law, a beryllium foil of 150 μm thickness attenuates the average energy of 1.25 MeV gamma rays by only 0.01%, necessitating consideration for the treatment of outgoing gamma rays. These rays are predominantly unattenuated gamma rays that have not interacted with the beryllium foil, with a small fraction being scattered gamma rays. The outgoing gamma rays, which have suffered minimal loss, are highly useful for other imaging experiments or for the addition of measurement devices, such as Compton spectrometers, to cross-validate our system’s capabilities. The proposed system has taken this into account, employing an off-axis structure with a flange interface for future experiments. However, the primary goal of this experiment is to validate the system design. Thus, a 100-micrometer-thick titanium vacuum blind flange was placed at the flange interface to seal the vacuum, with its thin profile reducing the generation of scattered electrons and thereby minimizing background noise in the image. The experiment accounted for the impact of electrons and scattered gamma rays produced by the interaction of outgoing gamma rays with the titanium baffle on the image. To shield these scattered particles, a wall constructed from lead bricks was utilized. As for the majority of gamma rays that pass through the titanium baffle, thanks to the spacious laboratory, a lead-brick-lined beam dump was positioned approximately 15 m away to collect and attenuate these gamma rays, reducing their adverse impact on imaging.

## 4. Results and Discussion

### 4.1. Demonstration of Imaging Performance and Assessment of Spatial Resolution

**Figure 5:**
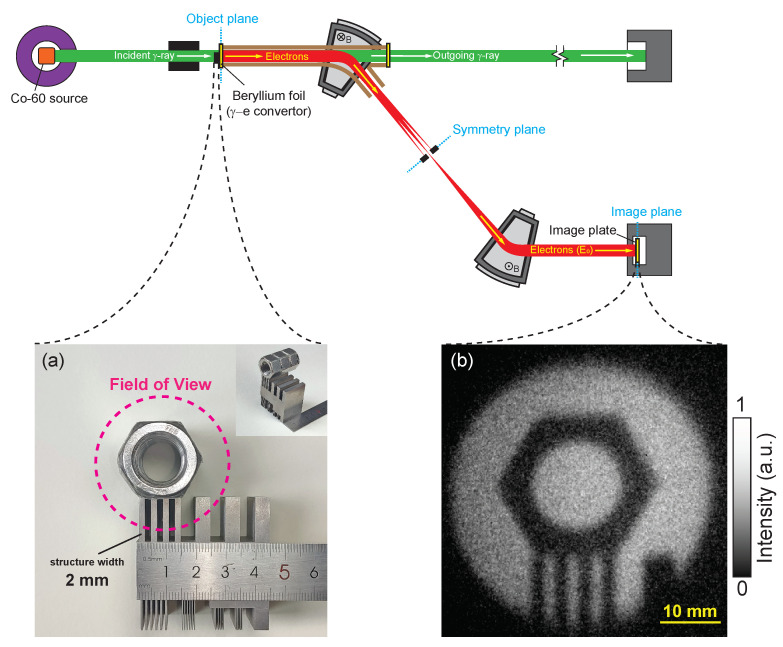
(**a**) Photograph of the object for gamma radiography. (**b**) Imaging results of the proposed system on the object.

The gamma-ray radiography method was employed to demonstrate the imaging effects. An object, as shown in Figure 5a, was positioned in front of the conversion target to attenuate the gamma rays’ intensity. The object for imaging comprises a tungsten line-pair array and three M16 nuts. The 40 mm field-of-view gamma-ray beam illuminates a segment of the tungsten line pairs, each 2 mm wide and 30 mm thick along the gamma-ray’s trajectory. Tungsten has a density of approximately 19.35 g/cm^3^. The nuts are made of 304 stainless steel, which has a density of 7.93 g/cm^3^. With an inner diameter of 16 mm and a thickness of 14.8 mm, the nuts are aligned and superimposed along the gamma-ray’s path. According to Lambert–Beer law, gamma rays with an average energy of 1.25 MeV are attenuated by 96% through the tungsten and 85% through the nuts. The attenuated gamma rays then strike the beryllium target, leading to the production of conversion electrons. These electrons are subsequently guided into the magnet system for imaging purposes. The target gamma-ray energy for the magnet system was set to 1.332 MeV, employing an irradiation duration of 30 min. The electron image on the image plane, as recorded by the imaging plate, is presented in Figure 5b. The findings confirm that the proposed system is effective for imaging gamma rays’ incident on the beryllium target and can clearly resolve line-pair structures of 2 mm in width, with no significant image distortion observed.

**Figure 6:**
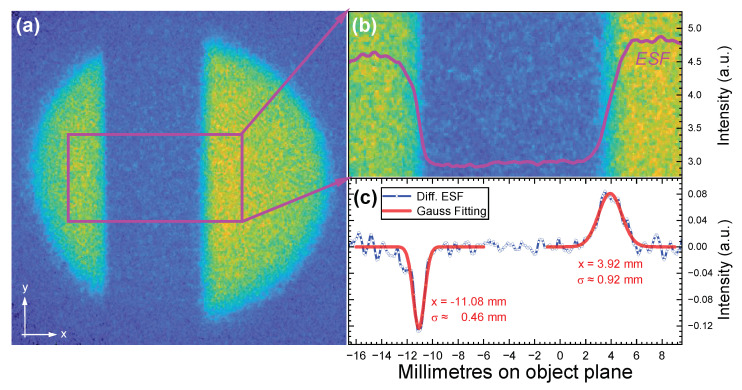
Quantitative assessment of spatial resolution for tungsten block imaging. (**a**) Imaging results of the tungsten block. (**b**) Magnified image of the rectangular region in (**a**) and intensity projection in the x-direction, i.e., edge spread function along the edge of the tungsten block. (**c**) Calculated line spread function (i.e., the derivative of the edge spread function) and its Gaussian fitting function.

The spatial resolution of the system was quantitatively evaluated using the knife-edge method [22]. A 40-millimeter-thick tungsten block was positioned in front of the conversion target to attenuate the gamma rays along their direction of incidence. With consistent parameter settings, the resulting images are presented in Figure 6a. The well-defined edge of the tungsten block was used to estimate the system’s spatial resolution. This involved measuring the electron intensity variation across the block’s edge to determine the edge spread function (ESF), as shown in Figure 6b. The sharpness of the image edge is inferred from the derivative of the ESF, which is the line spread function (LSF). A Gaussian function was then fitted to the LSF, as shown in Figure 6c. Given the known dimensions of the tungsten block, the imaging measurements can be calibrated to the actual object plane. After calibration, the 1σ resolution on the object plane was found to be approximately 0.46 mm at x=−11.08 mm and 0.92 mm at x=3.92 mm.

The experiments demonstrated the imaging process for 1.332 MeV gamma rays, confirming the system’s ability to correctly image gamma rays and validate the correctness of the beam optics design. The imaging results showed no significant distortion, and the system’s spatial resolution was assessed to be better than 1 mm using the knife-edge method. However, it should be noted that the results, as depicted in Figure 6, indicate spatial resolution variability across different locations within the system; the 1σ resolution on the object plane was approximately 0.46 mm at x=−11.08 mm and 0.92 mm at x=3.92 mm. This variability may be attributed to several factors:

1. Inaccuracies in the experimental setup, particularly the alignment of the two large and heavy magnets (each weighing over 300 kg), which can lead to misalignment and disrupt the imaging conditions, resulting in aberrations as shown in Figure 6.
2. Imprecise placement of the tungsten block used for imaging; for instance, if the block is tilted, it can cause inconsistent attenuation of gamma rays along its edges, leading to the observed results in Figure 6.

It should be emphasized that achieving high spatial resolution and high detection efficiency simultaneously is not feasible, and a trade-off is necessary. The enhancement of spatial resolution often comes at the expense of detection efficiency. Typically, system structural parameters are selected based on practical applications to optimize system performance. Nevertheless, the imaging results, obtained within a 40 mm field of view, are encouraging and indicate the potential application value of this imaging system in specific domains.

### 4.2. Evaluation of Energy Resolution and Detection Efficiency

**Figure 7:**
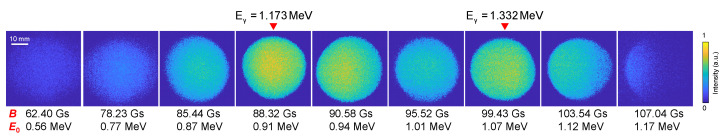
Imaging results of circular gamma ray beams under different magnetic fields.

By varying the current of the magnets, the central magnetic flux density *B* can be adjusted, thus enabling the imaging of gamma rays with varying target energies. In our experiment, a gamma-ray beam, approximately 40 mm in diameter, was collimated and directed onto the beryllium target. Irradiation was performed under different magnet current settings, with a duration of 30 min for each exposure. The electron images, captured by the image plate under various central magnetic flux densities *B*, are presented in Figure 7. A direct correlation is observed between the magnetic flux density *B*, the average energy $E_{0}$ of the electrons at the image plane, and the energy of the incident gamma rays, which is also indicated in Figure 7.

The presented results indicate that increasing the magnetic flux density *B* leads to a significant alteration in image intensity. This variation underscores the imaging system’s differential response to gamma rays with unique energy levels. The Co-60 source is known to emit gamma rays at energies of 1.173 MeV and 1.332 MeV. Calibration of the magnetic flux density *B* to correspond with the energies of the recoil electrons produced by these gamma rays results in electron images on the imaging plane with the most pronounced grayscale values. Concurrently, the gamma-ray beam are at their most discernible and well-defined. This observation serves as a compelling validation of the system’s capability for energy-selective imaging of gamma rays.

**Figure 8:**
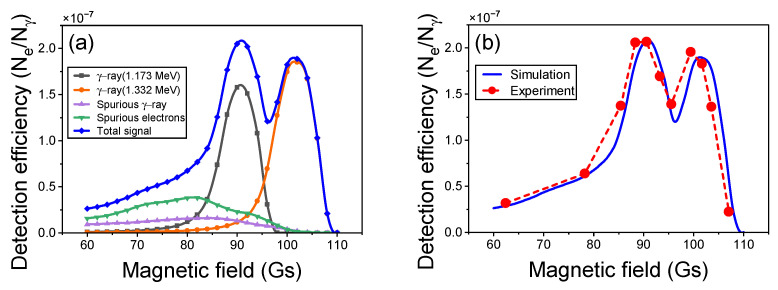
The detection efficiency of the system for Co-60 radiation imaging under different magnetic fields. (**a**) Monte Carlo simulation results based on Geant4. (**b**) Comparison of experimental and simulation results.

By integrating the grayscale values of images captured across a range of magnetic flux density and applying Equation (3), we convert these values into PSL. Utilizing the calibration data available for our imaging plate and scanner model, we then translate PSL into the number of electrons $N_{e}$ arriving at the imaging plane. With the knowledge of the gamma photon flux and the irradiation duration as detailed in Table 1, we calculate the incident gamma photon beam $N_{\gamma }$ that strikes the beryllium target. This calculation enables us to determine the system’s detection efficiency at various magnetic flux density for the Co-60 radiation source. Employing identical parameter settings, we simulated the entire system using the Geant4 Monte Carlo simulation software to compute the detection efficiency across different central magnetic fields *B*. The simulation outcomes, featured in Figure 8, demonstrate an excellent agreement with the experimental data.

As illustrated in Figure 8, the system’s detection efficiencies for 1.173 MeV and 1.332 MeV gamma rays were determined to be $1.58\times 10^{-7}$ and $1.86\times 10^{-7}$, respectively. In comparison, the Compton spectrometer [14,15,23], which also detects MeV-energy gamma rays using Compton electrons, exhibits a detection efficiency of approximately $10^{-4}$, higher than that of our system. The efficiency discrepancy between the two systems primarily stems from two factors:

1. Our system achieves gamma-ray energy-selective imaging by recovering images from Compton electrons, employing a collimator to improve spatial resolution at the expense of some detection efficiency because of electron divergence and energy spread.
2. The Compton spectrometer, designed for gamma-ray spectral analysis, forgoes collimators for higher detection efficiency, accepting some image blurring from electron dispersion.

The experimental results validate the correctness of our design. However, the current system’s detection efficiency is relatively low for practical applications, which limits its broader use. Enhancing detection efficiency will be a focal point of our subsequent work. We plan to improve spatial resolution through the following approaches:

1. Optimizing beam optics. Experiments have confirmed that the structure, composed of two dipole magnets, can recover gamma-ray image information. Fine-tuning the parameters of the magnets and collimators can enhance the system’s imaging capabilities, allowing gamma rays with larger divergence angles and energy dispersion to reach the image plane without compromising spatial resolution, thereby reducing beam loss and enhancing detection efficiency.
2. Improving hardware design. The current prototype’s hardware components have not been fully optimized. For example, the vacuum channel system, which provides a vacuum environment for electron transport, may not offer sufficient space for electron movement, leading to unnecessary collisions and beam loss.
3. Incorporating image reconstruction algorithms. Since the imaging process of our system can be accurately modeled using Monte Carlo simulations, the theoretical point spread function can be simulated. This provides a basis for introducing image reconstruction algorithms, which can reduce the system’s reliance on beam optical design, enabling a trade-off between spatial resolution and higher detection efficiency.

## 5. Conclusions

This paper introduces a novel gamma-ray imaging system that capitalizes on forward-scattered Compton recoil electrons. The system’s design is concise, employing only two dipole electromagnets and collimators to control electron trajectories, thus achieving indirect energy-selective gamma-ray imaging. Validated through experiments, the prototype has demonstrated precision in recovering the spatial distribution of gamma rays and in energy-selective discrimination. A key feature is its adjustable energy selection, which extends its potential applications across diverse fields. Despite these advancements, there is scope for improving detection efficiency and spatial resolution. Future research will focus on optimizing beam optics, enhancing hardware, and integrating advanced image reconstruction algorithms. This study establishes a solid foundation for the advancement of gamma-ray detection technology.

## Figures and Tables

**Table 1:** Parameters of the gamma-ray beams hit on the convertor.

Parameter	Value
Energy of the photons	1.173 MeV, 1.332 MeV
Distance from source to convertor	1.5 m
Diameter of the beam after collimation	40 mm
Dose rate at the convertor	49.84 R/min
Flux at the convertor	1.36 × $10^{9}$ γ/(cm^2^ · s)
Exposure time	30 min

## Data Availability

The data that support the findings of this study are available from the corresponding author upon reasonable request.
